# Effects of altered salt intake and diet on cytokines in humans: A 20‐week randomized cross‐over intervention study

**DOI:** 10.1002/eji.202250074

**Published:** 2022-11-20

**Authors:** Teemu Niiranen, Iris Erlund, Sirpa Jalkanen, Antti Jula, Marko Salmi

**Affiliations:** ^1^ Department of Medicine Turku University Hospital and University of Turku Turku Finland; ^2^ Department of Public Health Solutions Finnish Institute for Health and Welfare Helsinki Finland; ^3^ Department of Government Services Finnish Institute for Health and Welfare Helsinki Finland; ^4^ MediCity Research Laboratory University of Turku Turku Finland; ^5^ Institute of Biomedicine University of Turku Turku Finland; ^6^ InFLAMES Research Flagship Center University of Turku Turku Finland

**Keywords:** clinical trial, cytokines, diet changes, inflammation, salt intake

## Abstract

High sodium concentration alters leukocyte activation, and in particular T‐helper (Th) lymphocyte polarization, and drives the development of autoimmune diseases in mouse studies. Similar results have been obtained with human leukocytes under *in vitro* settings and in few observational studies. Therefore, salt has been implicated as a risk factor for autoimmune diseases. Here, we examined whether physiologically relevant changes in salt intake or diet alter cytokine concentrations. In a 20‐wk double‐blinded, placebo‐controlled study 106 participants were randomized to Habitual and Healthy Nordic diets, and further to Usual Sodium and Reduced Sodium intake groups using a cross‐over setup. Plasma concentrations of 45 cytokines were measured at three different time‐points using a multiplex assay. Repeated analyses of covariance revealed that high salt ingestion (or changes in the diet) did not induce significant changes in any of the signature cytokines controlling Th1, Th2 or Th17 polarization. Several other pro‐inflammatory interleukins, chemokines and growth factors were also unaffected by the level of salt intake or changes in the diet. We conclude that in humans clinically relevant changes in salt intake or diet do not have reflections on the systemic concentrations of pro‐inflammatory cytokines in vivo.

## Graphical abstract

The effect of habitual Western diet and salt‐reduced diet on the levels of systemic blood cytokines was analyzed in a placebo‐controlled nutritional intervention study. The results showed that reduced salt intake did not alter the concentrations of any of the analyzed 34 cytokines in blood. Our data show that marked real‐word changes in the diet can take place without any significant reflections on the systemic cytokine mileu in humans.

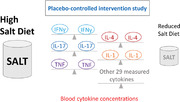

## Introduction

The nutritional and immune status are closely intertwined in humans [1, 2, 3]. Malnourishment is associated with impaired immune responses against microbes, whereas high‐fat diet induces a chronic low‐level inflammation in the body. Individual nutrients also have direct effects on the immune system. For instance, palmitic acid triggers Toll like receptor 2 activation, cytokine signaling and inflammation in the intestinal wall, vitamin A controls site‐specific activation of dendritic cells and phosphatidylcholine‐derived trimethylamine activates pro‐inflammatory macrophages [4, 5, 6]. On the other hand, carbazoles (from cruciferous vegetables), flavonoids and polyphenols promote the differentiation of anti‐inflammatory leukocytes and generation of anti‐inflammatory cytokines via triggering of aryl hydrocarbon receptors. Short chain fatty acids (generated from dietary fibers by bacterial fermentation) and omega‐3‐fatty acids promote anti‐inflammatory responses via other receptors [7, 8, 9, 10, 11].

Sodium is another nutrient which not only regulates the blood pressure, but is also thought to have direct pro‐inflammatory and autoimmune disease‐inducing effects on the immune system [12, 13, 14, 15]. In mouse studies high‐salt diet alters the development of different cytokine‐producing T‐helper types. High sodium intake enhances the polarization of naïve T‐helper cells into pathogenic T‐helper 17 (Th17) lymphocytes and drives the development of multiple sclerosis‐like pathology by activating p38/MAPK and serum/glucocorticoid‐regulated kinase1 signaling pathways [16, 17, 18]. High‐salt diet also induces cerebral hypoperfusion and subsequent cognitive impairment in mice by increasing the synthesis of IL‐17, the main effector cytokine secreted by Th17 cells [14]. In mouse studies Th2‐type, and possibly Th1‐type, inflammatory reactions are also enhanced by increased sodium concentrations, while suppressive functions of regulatory T‐cells are inhibited [19, 20, 21, 22, 23, 24, 25]. Similar salt‐induced changes in the polarization of human T‐helper cell subsets have been observed under in vitro conditions [14, 16, 17, 19‐22]. Therefore, increased dietary salt intake has been suggested to be an environmental risk factor for driving pro‐inflammatory lymphocyte differentiation, and consequently, for triggering autoimmune and vascular diseases [12, 13, 26]. High salt has also been shown to impair the differentiation of myeloid derived suppressor cells, monocytes, and macrophages into immune‐suppressive cell types, which can aggravate autoimmunity but have beneficial effects on antitumor immunity and in control of selected microbial infections [27, 28, 29, 30, 31, 32]. In other infections, salt‐induced suppression of neutrophil functions has instead led to aggravated disease course [33]. If excess salt intake exacerbated the development of inflammatory diseases, improved anti‐tumor immune responses or modulated anti‐bacterial immunity this would have a huge impact, since the majority of Western people constantly ingest salt in excess of the dietary recommendations [34].

No study, to our knowledge, has examined the effect of salt intake on cytokine concentrations in a placebo‐controlled, intervention study setup. Therefore, our aim was to utilize unique samples available from a dietary cross‐over intervention study to assess the effect of the salt intake (and diet) on systemic cytokine concentrations under clinically relevant settings *in vivo*.

## Results

### Salt intervention in a cross‐over setup is effective

**Figure 1:**
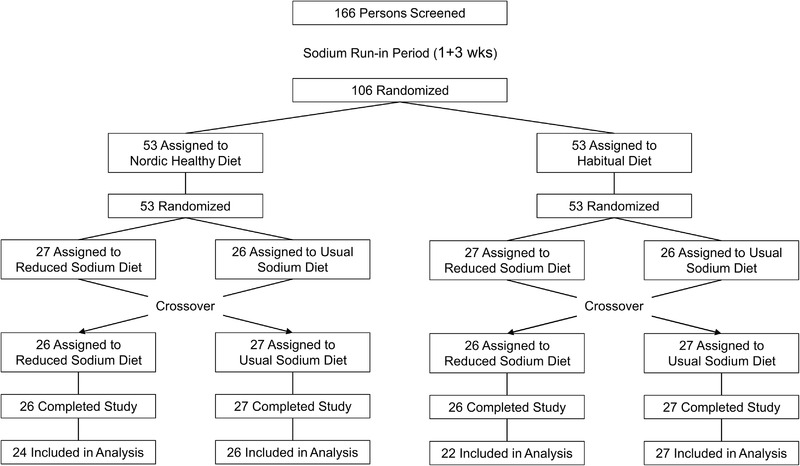
The study design. The numbers of participants included at each step of the study, the interventions (Healthy Nordic Diet vs. Habitual Diet and Usual Sodium Diet vs. Reduced Sodium Diet), and the timing of randomizations and cross‐overs are indicated on the flow chart.

In this double‐blinded, placebo‐controlled study 106 participants were randomized to Habitual and Healthy Nordic (berries, vegetables and fish enriched) diets, and further to Usual Sodium and Reduced Sodium intake groups (Figure 1). After eight weeks, a cross‐over of the Usual and Reduced Sodium groups was performed within both diet cohorts for additional 8 weeks (Figure 1). Baseline demographic and clinical characteristics of the participants are shown in Table 1.

**Table 1:** Baseline characteristics of participants by randomization group

Characteristic	Habitual Diet	Nordic Diet	Overall
Participants, n	49	50	99
Women, n (%)	27 (55.1%)	26 (52.0%)	53 (53.5%)
Age, mean (SD)	60.6 (7.9)	60.1 (8.2)	60.4 (8.0)
BMI, mean (SD)	26.5 (4.1)	26.1 (3.8)	26.3 (3.9)
Home systolic BP, mean (SD)	138 (15)	138 (14)	138 (15)
Home diastolic BP, mean (SD)	82 (8)	82 (8)	82 (8)
dU‐potassium (g), mean (SD)	3.3 (0.9)	3.2 (0.8)	3.3 (0.8)
dU‐sodium as salt (g), mean (SD)	10.2 (3.8)	9.7 (3.1)	10.0 (3.5)

Abbreviations: BMI, body mass index; BP, blood pressure; dU, daily (24‐hour) urinary.

**Figure 2:**
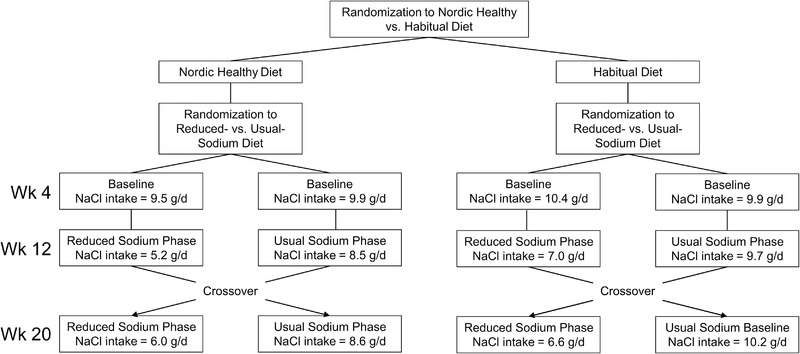
Twenty‐four hours urinary sodium excretion. The estimated dietary salt (NaCl) intake (mean of the indicated group) corresponding to the measured 24 h sodium excretion rates are shown at the baseline before randomization to different Salt diets (week 4), after the first salt intervention period (week 12) and in the end of the study (week 20) in the different groups. Blood samples for the cytokine measurements were drawn at the same time points.

The recommended sodium intake is below 2.3 g/d, which corresponds to 5.75 g salt [35], whereas the typical salt intake in Western populations is about 10 g/d [36]. In our study, the salt intake estimated by 24‐hour urinary sodium excretion was on average 10.2 g/d in the Habitual Diet group and 9.7 g/d in the Healthy Nordic Diet group after the 3 wk Sodium Run‐in Period at the time of randomization (Table 1, no significant difference between the groups). The sodium excretion decreased from randomization to the end of the Reduced Sodium intake period by 37.7% (corresponding to salt intake reduction from 9.94±3.5 to 6.19±3.0 g/d; p<0.001, Figure 2). No significant decrease from the randomization values was noted to the end of the Usual Sodium intake period (7.0% decrease, from 9.95 to 9.25 g/d; p = 0.06). After the cross‐over, we found that the sodium excretion in the Usual Sodium Diet → Reduced Sodium Diet groups decreased, while the sodium excretion in the Reduced Sodium → Usual Sodium Diet groups increased, as expected (Figure 2). These data indicate good adherence to the different salt intake regimens during the 20 wk study period.

### Performance of the multiplexed cytokine measurements

Out of the 45 cytokines measured, we excluded for the statistical analyses the 11 cytokines (IL‐1α, IL‐5, IL‐9, IL‐10, IL‐21, IL‐22, IL‐31, IFN‐α, TNF‐β, EGF, and VEGF‐D), in which >50% of the measurements remained under the detection limit of the multiplex assay (Table S1). Of the 34 cytokines included in the analyses, 20 had >95% of the measured values falling in the dynamic range of the assay (Table S1). The missing cytokine values for the 34 cytokines were imputed (see Materials and methods). Altogether 10.6% of the cytokine values were derived from the imputation. The intra‐assay and inter‐assay variation %CVs were on average 8.8 ± 1.9 and 13.6 ± 2.2 (mean ± SEM of all individual cytokine %CVs), respectively.

### High salt intake does not alter cytokine levels

**Figure 3:**
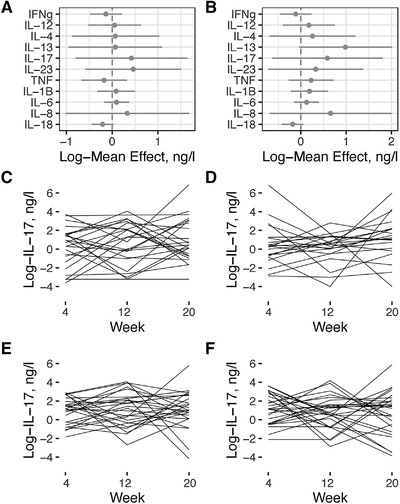
The effects of diet interventions on pro‐inflammatory interleukins. The effect of (**A**) Healthy Nordic vs. Habitual Diet and (**B**) Usual Sodium vs. Reduced Sodium Diet on the indicated plasma cytokine concentrations was determined using multiplex assays and repeated analyses of covariance tests. The results are presented as the mean ± SE with 95% confidence intervals of log‐transformed concentrations from the whole cohort. None of the changes were statistically significant (repeated analyses of covariance). (**C and D)** The concentrations of plasma IL‐17 at study weeks 4, 12, and 20 in individual participants (each line represents one individual) undergoing changes from Reduced Sodium diet to Usual Sodium Diet within (**C**) the Healthy Nordic Diet and (**D**) Habitual Diet groups. (E, F) The concentrations of IL‐17 at study weeks 4, 12, and 20 wk in individual participants (each line represents one individual) undergoing changes from Usual Sodium to Reduced Sodium Diets within (**E**) the Healthy Nordic Diet and (**F**) Habitual Diet groups. IFN, interferon, IL, interleukin.

**Figure 4:**
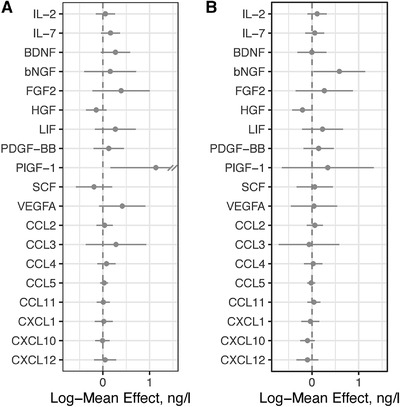
The effects of diet interventions on systemic cytokine concentrations. The effect of (**A**) Healthy Nordic vs. Habitual Diet and (**B**) Usual Sodium vs. Reduced Sodium Diet on the indicated plasma cytokine and growth factor concentrations was determined using multiplex assays and repeated analyses of covariance tests. The results are presented as the mean±SE with 95% confidence intervals of log‐transformed concentrations. None of the changes were statistically significant (repeated analyses of covariance). TNF, tumor necrosis factor; IFN, interferon; CCL, CC‐family chemokine; CXCL, CXC‐family chemokine; bNGF, brain neural growth factor; EGF, epidermal growth factor; FGF‐2, fibroblast growth factor‐2; HGF, hepatocyte growth factor; LIF, leukemia inhibitory factor; SCF, stem cell factor; BDNF, brain‐derived neurotrophic factor; GM‐CSF, granulocyte‐macrophage colony stimulating factor; PDGF‐BB, platelet derived growth factor‐BB; PlGF1, placental growth factor; VEGF, vascular endothelial growth factor.

At the time of randomization to Healthy Nordic Diet and Habitual Diet there were no significant differences in the level of any tested cytokine between the two groups (data not shown) verifying the validity of the randomization. The cytokine measurements at the time of cross‐over and at the end of the study showed that Reduced Sodium Diet vs. Usual Sodium Diet did not have any significant effect on the circulating cytokine concentrations. Notably, the Usual Sodium Diet did not increase any of the measured signature cytokines for Th1 (IFN‐γ, IL‐12), Th2 (IL‐4, IL‐13), or Th17 (IL‐17, IL‐23) lymphocytes (Figure 3A,B). Of particular note, the change in salt intake did not have any consistent effects on the concentration of IL‐17, the signature cytokine of Th17 lymphocytes (Figure 3C–F). There was also no difference in more general pro‐inflammatory cytokines (TNF, IL‐1β, IL‐6, IL‐8, IL‐18, TNF, IL‐18), which are typically produced by macrophages (Figure 3). The levels of lymphocyte and myeloid cell attracting chemokines (CCL2, CCL3, CCL4, CCL5, CCL11, CXCL1, CXCL10, and CXCL12) and growth factors regulation the production of leukocytes, endothelial cells and other cell types (IL‐2, IL‐7, SDF‐1, BDNF, bNGF, FGF2, HGF, LIF, PDGF‐BB, PlGF‐1, SCF, and VEGFA) were also not altered by the salt intake (Figure 4). Thus, under a controlled, physiologically relevant cross‐over setting, changes salt intake did not induce significant changes in systemic concentrations of any of multiple cytokines.

### Similar cytokine concentrations during the different diet interventions

We finally determined weather berries, vegetables and fish enriched Healthy Nordic Diet would exert anti‐inflammatory effects on the cytokine levels. Analyses of the samples from the three time points (baseline, cross‐over and end of the study) showed that the Healthy Nordic Diet did not significantly affect the concentrations of Th‐signature cytokines, pro‐inflammatory cytokines, chemokines or other cytokines (Figures 3 and 4).

## Discussion

The results from our clinical study show that the diet (fish and vegetable enriched Healthy Nordic Diet) or reduced salt intake does not change the levels of circulating cytokines in humans. We find the placebo‐controlled cross‐over study design, objective measurement of sodium excretion and relatively long duration (8+8 wk interventions) as strengths of our study. The provocative results on the salt‐induced Th2 and Th17 expansion, and their association with aggravated autoimmune reactivity in mice, thus seem not to be directly translatable into humans under physiologically relevant in vivo settings.

The reasons for the discrepant results between our study and those using mouse models may include genuine species‐specific differences in electrolyte metabolism and immune cell activation. Moreover, in the in vivo mouse experiments the animals have been suddenly exposed to chow, which contains 4–5% salt [14, 16, 17, 21, 22]. This is 8–16× more than laboratory mice normally ingest. In humans, in contrast, typical sodium ingestion in Western countries equals to 8.7–10.1 g of salt per day [36], which corresponds well to the sodium excretion values we measured in our study. Our Usual Salt Diet caused about 1.6‐fold increase in salt intake in comparison to Reduced Salt Diet, which is strikingly different from the massive salt loading used in mice models. When human lymphocytes were shown to polarize to Th17 direction in a salt‐inducible manner in vitro, a high salt concentration together with exogenous, Th polarizing cytokines was needed [14, 16, 17, 21, 22]. Notably all these previously used in vitro and in vivo conditions are clearly different from our current in vivo intervention trial.

A few studies have reported salt‐induced changes in cytokine secretion in humans under in vivo settings. In a spaceflight stimulation study (an enclosed habitat) six healthy males were subjected to fixed salt intake (daily doses of 12 g, 9 g, and 6 g salt; about 50 days under each condition) in a context of a very carefully controlled diet. Analyses of plasma TNFα, IL‐6, IL‐10 and IL‐17 did not reveal statistically different changes during the different salt ingestion phases [37]. In another study with eight volunteers, a 6 g/d increase in salt ingestion (estimated mean total salt intake 13.8 g/d) for 2 weeks resulted in a minor increase in Th17 positive cells in an open label setting study (using one tailed t‐test) [22]. Similarly, an increase in plasma IL‐17 was reported when a 7 day diet with very low salt intake (3 g/d) was switched into a very high salt intake (18 g/d) for another week [38]. Of note, both studies were open‐label and the compliance of the study subjects with the different salt intake was not verified using quantitative laboratory analyses. Collectively, our study and the previous studies imply to us that although acute, extremely high salt exposure may induce detectable changes in cytokine concentrations, physiologically relevant changes in the long‐term diets are unlikely to provoke a pro‐inflammatory cytokine profile in humans.

Mediterranean diet is consistently found to lower the systemic levels of pro‐inflammatory biomarkers [39], while the effects of Healthy Nordic Diet on inflammatory mediators have not been studied as thoroughly. The two diets have several common features, including increased use of fruits, vegetables (and berries in the Healthy Nordic Diet), whole‐grain products and fish, and restrictions in the use of saturated fats and red meat. In two large observational studies (altogether >6400 participants), Healthy Nordic Diet associated with lower hs‐CRP concentrations, while no changes in plasma TNF and IL‐6 concentrations were reported [40]. In a randomized controlled dietary trial, CRP, but not TNFα, decreased in the Nordic Diet group [41]. In two other controlled trials (NORDIET and SYSDIET), the changes in CRP were not reproduced [42, 43]. In the SYSDIET study, increase in IL1Ra concentrations was found, whereas the concentrations of the other measured cytokines (IL‐1β, IL‐6, IL‐10) were not altered [43]. Findings in our current cross‐over intervention study with an extended cytokine panel are consistent with the earlier analyses in suggesting that Healthy Nordic Diet does not cause any major alterations in the systemic concentrations of inflammatory cytokines.

Our study has several limitations. It includes a relatively small and heterogeneous group of mildly hypertensive individuals. The power calculations were done for the primary end point (blood pressure) and therefore higher number of participants would have increased the power of several cytokine analyses, although the cross‐over setup (each individual serving as her/his own control) is a clear strength in our study. Moreover, we cannot exclude the possibility that longer (or shorter) alterations in salt or diet would have altered cytokine levels. However, in other studies other types of dietary interventions have resulted in detectable alterations in cytokine levels in less than 8 weeks (e.g., [44, 45, 46]) indicating the duration of our study is at the physiologically relevant range. Moreover, we were not able to reach truly Low Salt –diet, which is typical challenge of real‐world Western intervention studies, and therefore it remains possible that the cytokine levels in our Reduced Salt groups are already induced by the salt levels which are higher than in most non‐Western populations. In addition, we only had fasting plasma samples available for measuring the soluble cytokines. Moreover, salt can be stored at higher concentrations in extrarenal tissues, where it is bound to the glycosaminoglycans in the extracellular matrix [32, 47].Therefore, it remains possible that salt or diet would induce detectable changes in local cytokine levels in peripheral tissues. However, it is of note that while an association between salt intake and activity of multiple sclerosis has been reported [26], other studies have failed to confirm it [48, 49].The production of proinflammatory cytokines by human in vitro cultured monocytes/macrophages is reported to be affected by sudden exposure to high salinity [50]. In our study setting it is impossible to determine which cell types (T‐lymphocytes, macrophages, others) contribute to the secretion of soluble cytokines, and if a certain cell type would respond to high sodium diet. Moreover, blood leukocytes were not collected from the participants, and therefore we were unable to analyze cytokine levels within T‐helper lymphocytes. Finally, the gut microbiota may have an essential role in mediating salt‐triggered changes in cytokine excretion [22, 51], but we do not have the possibility to study salt and diet induced changes in the intestinal microbiota.

In conclusion, our data suggest that increased salt intake at the levels seen in everyday life do not affect systemic cytokine levels in humans. Therefore, salt induced changes in cytokines are unlikely to induce the differentiation of pathogenic Th2 and Th17 responses in humans. These observations have important implications in immunology and medicine, since they do not support the literature proposing that increased salt intake might precipitate the emergence of autoimmune disease or that restricting salt intake would be a therapeutic option for treating autoimmune disorders.

## Materials and methods

### Participants and the design of the trial

Previously untreated mildly hypertensive men and women (45‐77 years) living in Southwestern Finland were screened from individuals, who had earlier participated in a national FINRISK 2007 health survey [52]. Potential participants were invited for briefing about the study, for giving their informed consent and for screening.

The subject could be included in the study if mean resting systolic blood pressure was 140–160 mmHg or a diastolic blood pressure was 90–100 mmHg. The participants filled in questionnaires for medical history. Individuals who were vegetarians or smoked, or with regular medication or vitamin use, cardiovascular disease, diabetes, gastrointestinal disease, fish allergies, a body mass index higher than 35 kg/m^2^, or excessive alcohol use were excluded from the study. A sample size of 109 was calculated with the assumption that a difference of 5 mm Hg (SD 9.3 mm Hg) change in systolic blood pressure can be detected with 80% power and 5% type I error (n = 88) for the whole study cohort as in [53]. The primary outcome was the change from baseline in systolic and diastolic blood pressure after treatments (to be reported separately), and secondary outcomes included change from baseline in biomarkers of cardiovascular disease risks (from which cytokines are reported here).

Participants included in the study first entered a 1‐week period during which they practiced maintaining a low sodium diet with a goal of 5 grams of salt per day (Figure 1). At the end of this period, 24‐h urinary sodium excretion was measured. Based on the results, each participant was given a unique number of sodium tablets to raise his/her salt intake to a high level (10 g/d) for the following 3 weeks. After this period, the participants were randomly allocated to a Healthy Nordic Diet or a Habitual Diet group (see below) at week 4. In both groups, a second randomization was performed, and the subjects received sodium tablets to meet a salt intake of 10 g/d (Usual Salt Diet) or matching placebo to meet a salt intake of 5 g/d (Reduced Salt Diet) for 8 weeks. Thereafter, a cross‐over between the Reduced Salt Diet and Usual Salt Diet was performed, and the participants continued in these new sodium diet phases to the end of the study period (for another 8 weeks) (Figure 1). A total of 99 subjects completed the study. The whole study was performed in a double‐blind fashion with quadruple masking (i.e. the participant, care provider, investigator and outcomes assessor were unaware of the intervention groups).

The study flow chart (CONSORT 2010) is given in Figure S1. The study was conducted according to the latest revision of the Declaration of Helsinki and was approved by the Ethical Committee of the Social Insurance Institution of Finland. The trial was registered at ClinicalTrials.gov as NCT01412346 (Health effects of Nordic diet rich in plant‐based foods and fish).

### Measurements and cytokine analyses

Blood pressure, weight, and height were measured, and 12‐hour fasting blood samples were taken before randomization at the screening visit (0 weeks) and at the 4‐, 12‐ and 20‐week visits. EDTA‐plasma was separated from the blood using standard procedures. In addition, a 24‐hour urine collection was performed before the 1‐, 4‐, 12‐ and 20‐week visits. Plasma and urine samples were frozen and stored at –70°C until assayed.

Daily urinary sodium excretion was determined using an ion‐selective electrode (Optima analyzer, Thermo Electron Oy, Vantaa, Finland). Based on the measured 24 h urinary sodium (U‐Na) excretion rate we calculated the estimated daily salt (sodium chloride, NaCl) intake using a formula NaCl (g/24h) = U‐Na (mmol/24h)x58.44×0.001.

The concentrations of 45 cytokines were measured from previously unthawed plasma samples using antibody‐based magnetic bead kits (Procarta Plex, Thermo) for multiplexed protein quantification according to the manufacturer's instructions. The cytokines analyzed were: interleukins (IL) IL‐1α, IL‐1β, IL1‐RA (receptor antagonist), IL‐2, IL‐4, IL‐5, IL‐6, IL‐7, IL‐8, IL‐9, IL‐10, IL12p70, IL‐13, IL‐15, IL‐17A, IL18, IL‐21, IL‐22, IL‐23, IL‐27, IL‐31, interferons (IFN) IFN‐α, IFN‐γ, tumor necrosis factors TNF‐α, TNF‐β, chemokines CCL2, CCL3, CCL4, CCL5, CCL11, CXCL1, CXCL10, and CXCL12, and growth factors bNGF (nerve growth factor beta), EGF (epidermal growth factor), FGF‐2 (fibroblast growth factor‐2), HGF (hepatocyte growth factor), LIF (leukemia inhibitory factor), SCF (stem cell factor), BDNF (brain‐derived neurothrophic factor), GM‐CSF (granulocyte‐macrophage colony stimulating factor), PDGF‐BB (platelet derived growth factor‐BB), PlGF1 (placental growth factor), VEGFA (vascular endothelial growth factor A), and VEGFD.

All measurements and analyses were done blinded to the intervention allocation of the subject. The baseline and follow‐up samples were always analyzed in one analytical run.

### Interventions

*Diet*: The subjects randomized to the Healthy Nordic Diet were given instructions to consume daily the following foods as part of their normal diet: a bowl of oatmeal porridge with berries every morning, four loafs of high‐grain rye or multigrain bread daily; rainbow trout patties (210 g) and cooked whole grain barley (1 dl) three times per week, and four bags of mixed frozen vegetables (3×200 g and 1×300 g) weekly. The foods were given to the subjects for free. Subjects randomized to the Habitual Diet group were advised to continue eating their usual diet during the study period. Compliance with the diet was followed using food consumption diaries.

*Sodium*: Each patient received his or her own bottle containing the sodium or placebo tablets for each study period. The amount of sodium was set so that each individual would receive approximately 10 g of salt per day during the Usual Salt phase and 5 g of salt per day during the Reduced Salt phase. Compliance with the drug treatment was controlled by counting the number of returned capsules, and by the 24‐hour urinary sodium excretion analyses.

### Statistical analysis

For the statistical analyses, we only included the 34 cytokines in which >50% of the measurements remained within the detection limit (Supplementary Table S1). To address missing data we imputed 5 complete datasets of cytokines at each sampling time point using the Markov Chain Monte Carlo method [54]. Our imputation model included sex, age, randomization group, sampling time point and all 34 cytokines. Log transformations were applied.

Baseline comparisons between the Healthy Nordic Diet and Habitual Diet groups were made with a *t* test for continuous variables and by a χ^2^ test for categorical variables to verify the success of the randomization. Using the statistical approach described in detail in Jula et al. [53], analysis of variance for repeated measures of variance, with contrasts between baseline and Usual Salt or Reduced Salt treatment periods, was used to test the significance of dietary changes within the Healthy Nordic Diet and Habitual Diet groups. The analysis of variance model was fitted separately to the Healthy Nordic Diet and Habitual Diet groups, where period and carryover effects were tested. Because no period or carryover effects were observed and baseline values affected the outcome, repeated analyses of covariance with baseline values as covariates, Healthy Nordic Diet and Habitual Diet as intersubject factors, and placebo and usual‐sodium treatment as intrasubject factors were included in the final models. Validity of the models was evaluated with residual analysis. Normality of residuals was checked with the Shapiro‐Wilk statistics and constancy of residuals by a graphic analysis.

The data are given as mean (SE) values with 95% confidence intervals for the mean changes. We used P = 0.001 as the level of significance to account for the number of cytokines (0.05 divided by 34). All statistical analyses were conducted with SAS version 9.4 (SAS Institute, Cary, NC).

## Conflict of interest

The authors declare no commercial or financial conflict of interest.

## Ethics approval statement for human studies

The study was conducted according to the latest revision of the Declaration of Helsinki and was approved by the Ethical Committee of the Social Insurance Institution of Finland. This work was supported by the Emil Aaltonen Foundation [to T.N.], the Paavo Nurmi Foundation [to T.N.], the Finnish Medical Foundation [to T.N.], the Finnish Foundation for Cardiovascular research [to T.N.] and Academy of Finland [321351 to T.N.; 129479 to I.E., 141136 to S.J. and M.S.].

## Patient consent statement

Informed consent was obtained from each participant of the study.

## Clinical trial registration

The study was registered at ClinicalTrials.gov as NCT01412346.

## Author contributions

I.E. and A.J. lead the intervention study. M.S. conceptualized the cytokine study. T.N., I.E., A.J., S.J., and M.S. performed investigation and data interpretation. T.N. performed statistical analyses. T.N. and M.S. wrote the manuscript. All authors have read and agreed to the published version of the manuscript.

### Peer review

The peer review history for this article is available at https://publons.com/publon/10.1002/eji.202250074

**Abbreviations**
CCL: C‐C chemokine
CXCL: C‐X‐C chemokine
GF: growth factor
IFN: interferon
IL: interleukin
Th: T‐helper
TNF: tumor necrosis factor

## Supporting information

Supporting Information

## Data Availability

The data that support the findings of this study are available on request from the Principal Investigators of the clinical study (A.J., I.E) pending on an application and approval by Finnish Institute for Health and Welfare. The data are not publicly available due to privacy or ethical restrictions.
