# Supplementary material for: Effects of altered salt intake and diet on cytokines in humans: A 20‐week randomized cross‐over intervention study
Source: Eur J Immunol. 2022 Nov 20;53(1):2250074. doi: 10.1002/eji.202250074 (PMC10100453; doi:10.1002/eji.202250074)

Supporting information for

Effects of altered salt intake and diet on cytokines in humans: a 20-week randomized cross-over intervention study

Teemu Niiranen [a,b], Iris Erlund [c,d], Sirpa Jalkanen [e,f], Antti Jula[b], Marko Salmi[e,f]

Supplementary Table 1. Performance of the cytokine measurements

Cytokine	% of in-range measurements	Median (25th-75th percentile)
IFNg	100	24 (17-33)
IL-18	100	115 (86-176)
LIF	100	12 (8-23)
CXCL1	99.7	58 (50-72)
BDGF-BB	99.3	718 (428-1176)
BDNF	99.3	615 (426-904)
CCL11	99.3	60 (48-71)
HGF	99.3	1091 (837-1389)
IL-1RA	99.3	1685 (1211-2234)
IL-1B	99.3	49 (30-70)
IL-6	99.3	40 (27-61)
IL-7	99.3	6 (5-8)
CXCL10	99.3	211 (173-262)
CCL2	99.3	142 (114-246)
CCL4	99.3	177 (153-220)
CCL5	99.3	456 (412-508)
CXCL12	99.3	1821 (1654-2055)
IL-2	99	50 (39-67)
VEGFA	99	179 (111-335)
SCF	98.7	49 (38-60)
CCL3	94.3	45 (20-144)
TNF	93.9	12 (7-18)
GM-CSF	91.2	55 (36-90)
FGF2	90.2	63 (40-97)
IL-12	87.5	3 (3-4)
bNGF	83.8	81 (55-116)
PIGF-1	76.8	28 (14-60)
IL-27	69.7	111 (54-281)
IL-13	69	3 (2-7)
IL-17	62.6	4 (2-17)
IL-8	62.3	7 (3-19)
IL-15	60.6	45 (23-86)
IL-4	58.2	13 (5-25)
IL-23	53.2	155 (53-437)
EGF	49.8	5 (1-10)
IL-5	34.7	14 (3-35)
IL-22	23.9	970 (401-3102)
IL-1A	22.2	16 (5-85)
IL-10	21.2	3 (0-14)
VEGF-D	18.9	23 (10-63)
TNF-B	17.8	107 (49-368)
IFN-A	17.2	2 (1-8)

IL-21	12.5	398 (91-775)
IL-31	12.5	87 (47-697)
IL-9	4.4	506 (284-1398)

Supplementary Fig. 1. CONSORT 2010 Flow chart

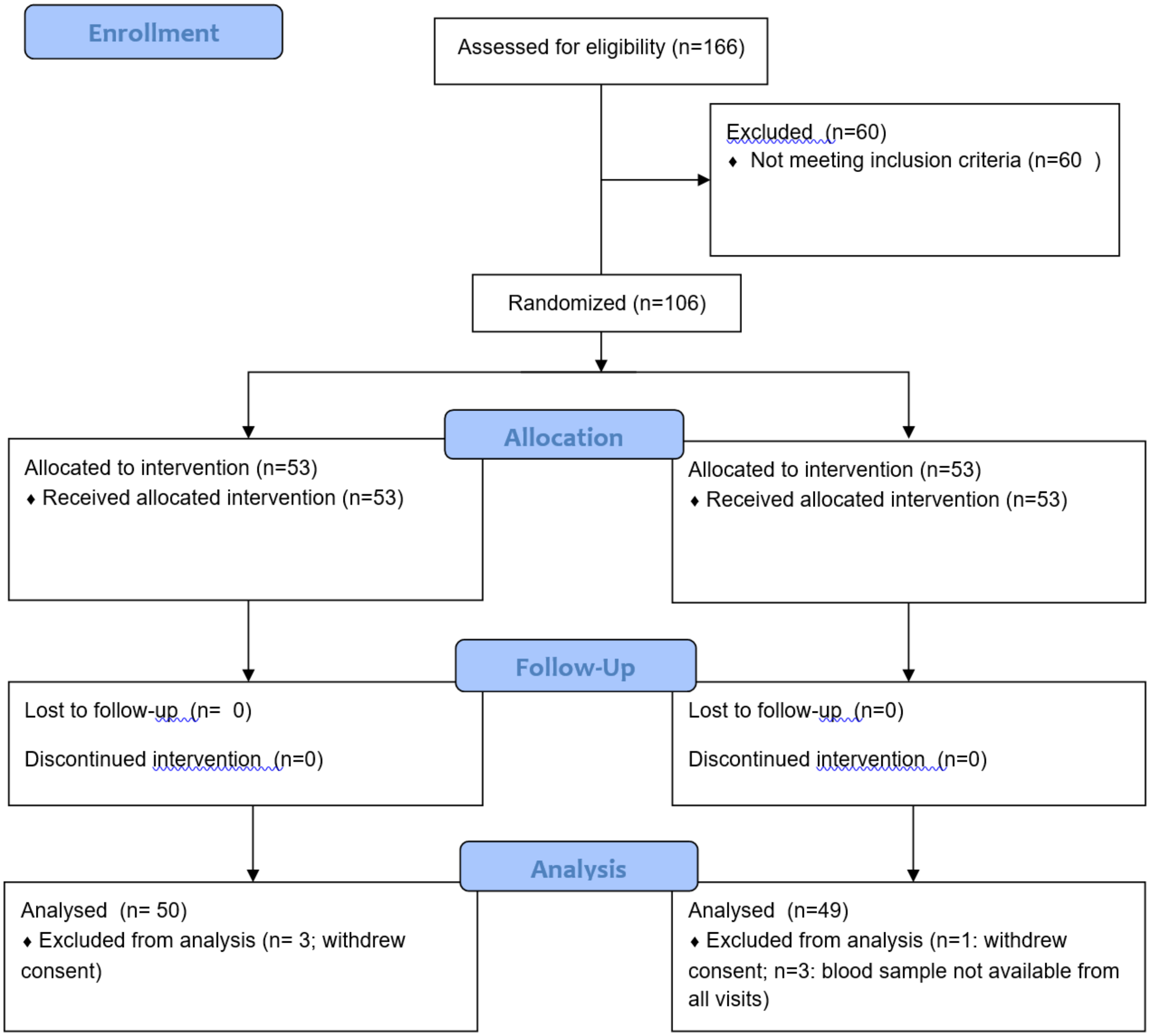

Supplement: Supplementary file 1 — Supporting Information [file EJI-53-0-s001.pdf]
